# Advances in Downstream Processing of Monoclonal Antibodies

**DOI:** 10.3390/antib15040063

**Published:** 2026-07-24

**Authors:** Michał Kołodziej, Dorota Antos

**Affiliations:** Department of Chemical and Process Engineering, Rzeszów University of Technology, Powstańców Warszawy 6 Ave., 35-029 Rzeszów, Poland

**Keywords:** downstream processing, capture, polishing, formulation

## Abstract

Although mAbs have great therapeutic potential, their use in medicine is currently limited by the high cost of their manufacturing. Significant developments in upstream processing technologies have caused downstream processing (DSP) to become the manufacturing cost-driver. DSP consists of a number of operations included in the capture, polishing, and formulation steps that contribute to excessive material and buffer consumption. This is particularly true for the capture and polishing steps, in which tedious and costly chromatographic operations are involved to ensure an adequate purity level of the medical product. The final formulation step also increases the burden of buffer consumption. This review focuses on those time- and material-consuming DSP operations and describes key issues and challenges related to their realization. In each of the steps, capture, polishing, and formulation, the platform processing approaches are presented as well as directions for their development. In addition, we present alternative nonchromatographic approaches that can potentially be used in the capture and polishing steps, such as precipitation or extraction. Furthermore, we describe mAb processing by crystallization, which can potentially serve as an alternative platform in both polishing and formulation steps.

## 1. Introduction

Currently, a significant increase in the share of monoclonal antibodies (mAbs) in the pharmaceutical market is being observed. In recent years, more than 200 mAbs–based drugs have been introduced to the market. In 2024 alone, 21 therapeutic mAbs were approved [1,2,3]. However, despite the growing demand for bioactive mAbs, the high manufacturing cost limits their widespread use in medicine [4,5,6].

Recent advances in upstream protein processing (USP) methods have enabled an increase in mAb harvest titers from 0.2 mg mL^−1^ to 27 mg mL^−1^ [7,8,9], which has led to increased efficiency and reduced costs of the initial manufacturing phase. This made downstream protein processing (DSP) a production bottleneck, accounting for up to 70 percent of total production costs [3,7,10,11,12]. This can be attributed to strict standards imposed by regulatory organizations regarding the purity of the final product; levels of certain impurities, such as host cell proteins (HCPs) and DNA, must be reduced to a few ppm or even ppb. Therefore, the DSP scheme should be properly designed to provide high purity and stability of the final product as well as appropriate yield. To ensure an adequate level of these performance indicators, contemporary DSP strategies are predominantly based on highly selective chromatographic techniques. Nevertheless, chromatography is the main cost driver of the DSP stage. It is caused by a high price of chromatography resins, e.g., protein A-based affinity medium, and a high consumption of buffers ranging from 30,000 to 2,000,000 L per batch [7,12,13,14,15]. Moreover, diffusion and capacity limitations result in low productivity of chromatography [14,16,17]. These limitations have led to growing interest in alternative purification methods, including precipitation, crystallization, and membrane-based techniques [7,15,18,19,20].

In this paper, we present a review of the mAbs purification methods, which contribute the most to the performance of DSP. We describe both the platform that is commonly utilized for capture and polishing stages of mAb processing, which is dominated by chromatographic techniques, and recent approaches involving alternative nonchromatographic methods such as precipitation and extraction. Finally, we report approaches used for the formulation of mAbs that include standard membrane-based techniques and an alternative method based on the crystallization process.

## 2. DSP Platform for mAbs Production

**Figure 1:**
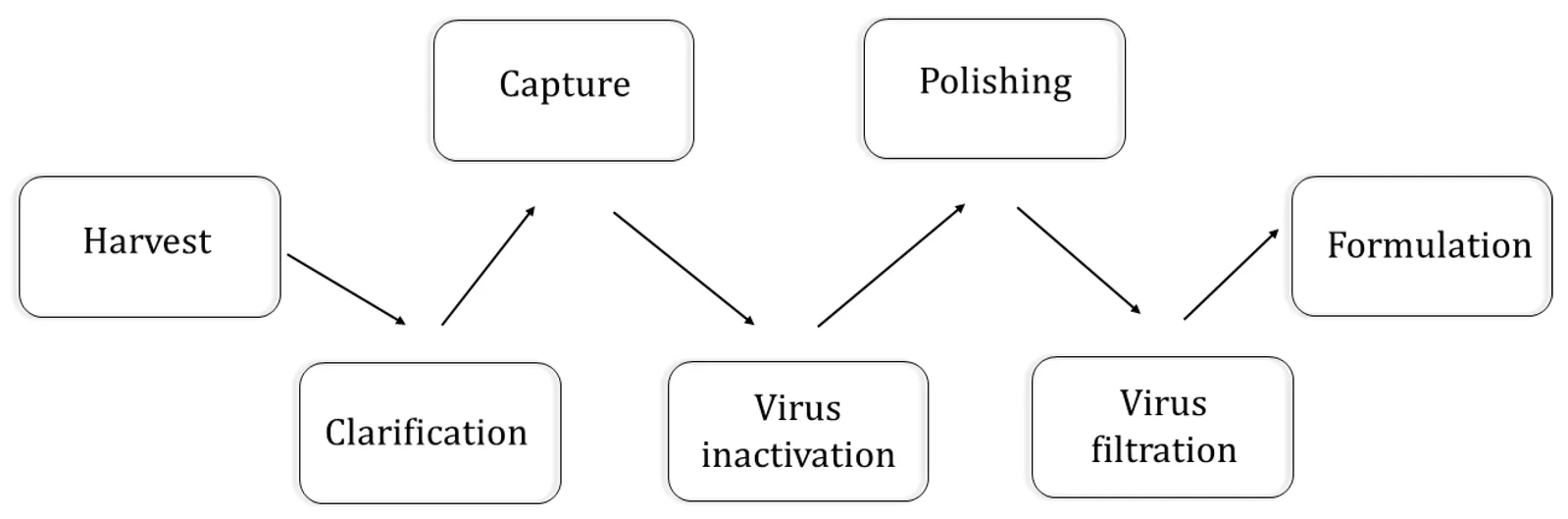
DSP flowsheet scheme typically used in mAbs production [27].

Although the DSP procedures used for the purification of mAbs have evolved over the last few decades, for the majority commercially produced mAbs, the DSP platform has remained the same (Figure 1). The bioreactor harvest material is first clarified by centrifugation and depth filtration. The protein was then isolated from the clarified solution in the capture step and subsequently subjected to low-pH treatment for virus inactivation, polishing, virus filtration, and formulation. Between these stages, additional buffer exchange operations are usually required [10,11,15,21,22,23,24].

The main challenge in developing a DSP procedure is the substantial diversity of monoclonal antibodies in terms of their physicochemical properties. Consequently, the particular process conditions are specific for the type of mAb and often prove unsuitable for another one [15,25,26]. Nevertheless, regardless of the mAb type, the capture, polishing, and formulation steps are the cost drivers in DSP. The standard and alternative techniques that can be used for realization of these steps, along with their advantages and disadvantages, are provided in Table 1.

## 3. mAb Capture Techniques

### 3.1. Platform Method

The capture step is typically accomplished by affinity chromatography (AC). The gold standard in the AC of mAbs is protein A chromatography (ProtA) in which a native or recombinant protein A ligand derived from *Staphylococcus aureus* (SpA) or *Escherichia coli* is used [28,29,30].

A typical procedure for ProtA capture includes loading the column with the clarified raw material, column washing at neutral pH, which is accompanied by impurity elution, and mAb elution by reducing the pH of the running buffer to 3–4 to weaken mAb–protein A interactions. Consequently, the ProtA elution step is most often coupled with a viral inactivation process, which also requires low pH treatment (Figure 1) [27,29,31].

The ProtA capture step provides mAbs isolated from process-related impurities such as HCP, host cell DNA, cell culture components, viruses transferred from biological material, and other contaminants [21,32,33]. However, it still may contain product-related impurities that exhibit affinity to ProtA resin, including mAb fragments, aggregates, charge variants, and post-translational modifications. It is also not completely free of process-related impurities that interact with adsorbed mAb molecules and coelute with them during the elution step. This explains the necessity for subsequent purification steps [21,34,35,36,37,38].

Commercially available ProtA resins provide a dynamic binding capacity (DBC) in the range of 40 to 120 g L^−1^. Manufacturers estimate the lifetime of most resins at 100 to 200 elution cycles [29]. In practice however, the DBC often decreases significantly after tens of purification cycles, which is caused by fouling ProtA ligands with impurities, such as HCP, whose residuals remain strongly bound to the resin during the elution stage. Their accumulation within the resin pores causes deactivation of the ligand and an increase in the column backpressure [29,39,40,41]. To mitigate fouling, the appropriate selection of regeneration conditions for the column cleaning-in-place (CIP) step is crucial. For that purpose, alkaline sodium hydroxide solutions are typically used, which allows the reduction of fouling as well as efficient resin sanitization [42,43]. However, repeated CIP cycles over time can trigger changes in the tertiary structure of protein A, thus affecting ligand affinity toward mAbs [29]. Therefore, current research is focused on the development of new, more stable chromatographic resins capable of withstanding higher NaOH concentrations, even up to 0.5 M [44,45,46,47]. Research efforts are also directed towards optimizing CIP conditions. For example, the application of acidic solutions with selected organic solvents is being investigated [48].

Despite these efforts, ProtA remains an economic bottleneck in DSP [6,7]. This is mainly due to the high cost of ProtA resins, which typically ranges between 8000 and 15,000 USD L^−1^ [49]. This inspires the search for new approaches for mAb capture by developing new less expensive affinity chromatography ligands that can substitute protein A, or non-chromatographic alternates that include precipitation or Aqueous Two Phase Extraction (ATPS) [7,28,29,50,51,52,53]. The advantages and disadvantages of these methods are summarized in Table 1.

### 3.2. Progress in AC Capture Methods

Currently investigated alternatives for ProtA include engineered SpA-mimicking ligands, biomimetic peptides, aptamers, and engineered small molecules [28,29,54,55]. For example, Wei et al. developed small peptide ligands, which allowed the isolation of IgG from Chinese hamster ovary cell culture (CHO) supernatants with purity exceeding 95% [56]. The developed resin was characterized by a relatively high DBC of 24.5 g L^−1^, but still distinctly lower than commercialized ProA resins. Another promising class of ligands is camelid single-domain antibodies (VHH). VHH-based resins exhibit mass transfer coefficients comparable to those of conventional ProtA resins. However, their main limitations include the lack of specificity toward antibodies derived from mouse, goat, and rabbit, as well as the requirement of low pH during elution (pH 2–3) [28,57,58].

Recently, protein G-derived calcium-dependent ligands have been developed for mild capture and elution of full-length IgG1 by interaction with their Fab fragment. The evolved ligand allowed calcium-triggered elution of the protein at neutral pH, which eliminated the need for low-pH treatment. This allowed the preservation of protein activity and structural integrity during elution [59,60,61,62].

In addition to ligand development, new support materials are also being explored. Beyond commonly used agarose, materials such as porous polymers, magnetic adsorbents, molecularly imprinted polymers, and nanomaterials have been investigated [17,28,63,64,65,66,67,68]. Particular attention is paid to porous polymer monoliths. These materials offer several advantages, including convective mass transport, weak flow-rate separation efficiency dependence, rapid separation, and low column pressure drop. Various ligands, such as proteins A, G, L, peptides, Con A, or L-histidine, can be immobilized onto the polymer matrix to enable specific binding of target proteins [69,70,71,72,73]. For example, Liu et al. used a boronate affinity monolith to capture IgG from human serum [74]. Du et al. isolated IgG from diluted human blood using a tentacle-type monolith immobilized with peptides, where the peptide ligand was attached to a glucose–GMA monomer [75]. Another class of matrix attracting significant interest include functionalized magnetic adsorbents. Magnetic field-assisted separation is a relatively fast and mild technique that helps preserve the full biological activity of the separated molecules. Since the magnetic core of these particles can trigger nonspecific adsorption, coating reagents, such as silylation agents, polymers, or surfactants, are typically applied prior to immobilization of the ligand [67,76,77,78]. Roque’s group achieved a high enrichment of human IgG (hIgG) from crude extracts using functionalized dextran coated magnetic particles. The binding capacity of hIgG exceeded 500 mg g^−1^, which is much higher than that of conventional ProtA. The purity of the final product reached 95% [78].

In addition to magnetic adsorbents and monoliths, functionalized membranes are increasingly popular for mAb capture. Because of their macroporous structure, they effectively reduce internal diffusion limitations. They are characterized by a relatively low backpressure, which allows operation at high flow rates, thus with a short protein residence time, which suppresses protein aggregation and denaturation [68,79,80]. For example, the Bruening group designed various membranes functionalized with mimotope peptides for the isolation of mAbs, including trastuzumab, bevacizumab, rituximab, and panitumumab. In the case of trastuzumab, the isolation of diluted human serum was completed in 5 min [80].

Recently, Berensmeier’s group has developed a new platform for mAb capture [81,82]. They used potential-controlled chromatography as an alternative to conventional low pH elution in the protein A membrane. In this approach, mAbs could be eluted from the affinity membrane at a mild pH and without necessity of buffer exchange [82]. Nevertheless, a very low ionic strength of the mobile phase is required for the successful processing of protein solutions, which may be problematic for the capture of mAbs from post-UPS mixtures of high ionic species contents.

A novel approach for eliminating low pH elution of mAbs is altering the dissociation equilibrium of protein A ligands by buffer composition. Rainer et al. showed that the proper combination of glycine buffer with a salt gradient triggers mAb elution at a mild pH of 6 [83].

**Figure 2:**
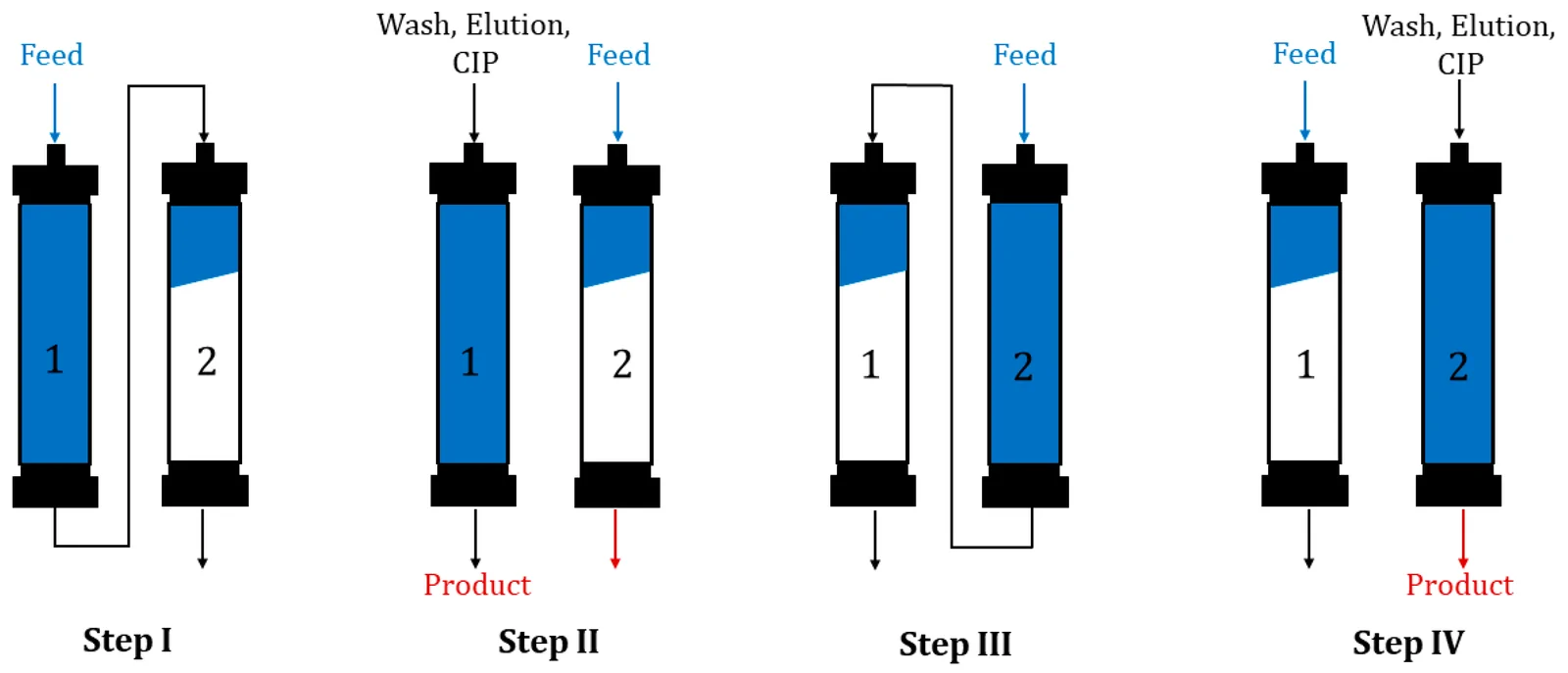
One cycle of ProtA with twin columns: in Step I, the feed is loaded onto column 1, while the effluent of this column is directed to column 2. Once the loading capacity is reached, the valves are switched, and the process moves to step II. During this step, column 1 is sequentially subjected to the wash, elution, and CIP stages, while the feed is simultaneously loaded onto column 2. Steps III and IV follow the same sequence as steps I and II, respectively, with the two columns exchanging their roles [90].

The progress in the mAb capture step has shifted toward continuous processing. The continuous mode of process realization is characterized by higher productivity and significantly lower consumption of buffers and chromatographic resin compared to conventional batch-wise chromatography [84,85,86,87]. Ötes et al. presented a downstream process of continuous ProtA based on end-to-end single-use systems, implemented for the manufacturing of a multispecific mAb. The developed process was characterized by a 17% reduction in buffer consumption and an 83% reduction in resin volume, resulting in an overall cost reduction of approximately 80% [87]. Capture SMB, also termed as twin-column ProtA, has also been shown to be a highly effective method for capture of mAbs. It is based on a two-column arrangement in which the columns operate alternatively in the loading or regeneration phases (Figure 2). It allows for better utilization of the resin capacity and increased productivity compared to batch processes [86,88,89].

However, continuous ProtA is not without limitations. A key limitation is the cycle-dependent decrease in process yield, coupled with a corresponding increase in the HCP content in the product [10,91,92].

The solutions presented in this section provide enhancement of the mAb capture efficiency and consequently reduce process costs. However, affinity-based capture techniques remain a cost-driver operation. Therefore, following the trend of ‘anything but chromatography’, alternative nonchromatographic methods are being explored for application in the capture stage [6,7,93].

### 3.3. Non-Chromatographic Capture Methods

Among the nonchromatographic methods that can be applied for mAb capture, precipitation is considered the most promising. In this process, polyethylene glycol (PEG) is commonly used as a precipitating agent. In a number of studies, PEG-aided precipitation has been reported to be an efficient method for selective capture of mAbs [12,19,51,93,94,95,96,97].

For example, Grosshans et al. successfully isolated commercially available mAbs using a combined precipitation and ion exchange chromatography process [98]. The yield of this process exceeded 96%, while a significant amount of impurities was removed. Hammerschmidt et al. developed a method for mAb purification from CHO harvest using a combined precipitation process that involved adjustment of pH and addition of PEG. In the first step, impurities were precipitated at reduced pH. Subsequently, after the pH was increased to the isoelectric point, mAbs were precipitated by the addition of PEG. The developed process was verified in continuous mode for three selected monoclonal antibodies [99,100].

Furthermore, several examples of hybrid processes of precipitation and membrane filtration can be found in the literature. Replacing centrifugation with membrane filtration for buffer exchange and subsequent precipitate redissolution enabled the development of single-use systems [19,96]. Such an approach was described by Minervini et al. who combined PEG-aided precipitation with continuous filtration for direct capture of mAbs from cell culture harvest. This process allowed the removal of a substantial fraction of HCP and DNA, at a product yield of approximately 92% [96].

**Figure 3:**
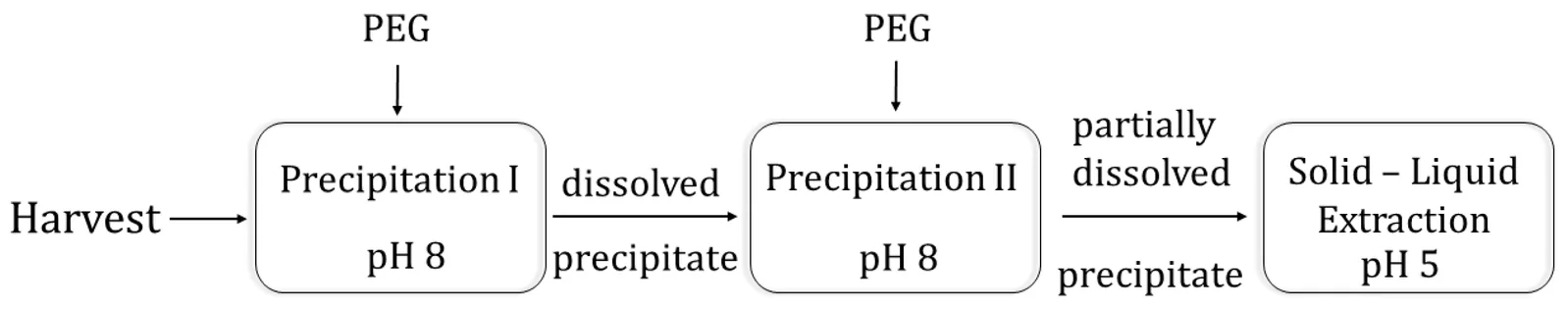
Scheme of the combined process of precipitation and solid–liquid extraction (SLE). In the first step, the mAb is precipitated from the harvest (pH 8), the precipitate is then redissolved and the precipitation is repeated under the same conditions to completely remove low-molecular weight impurities. In the last step of SLE, pure mAb is extracted from the precipitate (pH 5) [93].

In another approach, the mAb precipitation was combined with solid–liquid extraction [6,93]. In this process, two-stage precipitation was used to capture a mAb from a cell culture harvest in the presence of PEG solution of pH 8, which allowed the removal of low-molecular weight impurities. Next, the obtained precipitate was subjected to solid–liquid extraction (SLE) at reduced pH, at which pure mAb could be extracted from the solid phase while high-molecular weight impurities remained in the precipitate. In this approach, the extract contained the mAb with 99% chromatographic purity and a yield of 78%, with a DNA and HCP content reduced to 23 and 150 ppb. The process scheme is presented in Figure 3.

In addition to precipitation, aqueous two-phase extraction (ATPS) has also been applied as an alternative to the affinity-based capture step. Separation in ATPS is based on the differential affinity of mAbs and impurities toward two immiscible aqueous phases. Phase separation is achieved by mixing a polymer, most often PEG, with a suitable salt [50,101,102]. Several studies have reported the application of ATPS for the clarification and capture of mAbs [12,50,103,104,105]. For example, Kruse et al. described a DSP strategy in which both the clarification and ProtA stages were replaced by ATPS. The authors achieved mAb recovery yields of approximately 97% with final product purity greater than 95%. In addition, more than 99% of DNA and 97% of HCP were removed [50]. Achanta et al. developed a purification process for antibody-drug conjugates based on ATPS techniques. The separation efficiency of the method was further improved by centrifugal partition chromatography [102].

A continuous ATPS mode has also been investigated. Anupa et al. developed the continuous non-ProtA platform for mAb purification. They applied DOE-based optimization to integrate the ATPS process with HIC. The proposed approach provided the final product with a purity of 99.6% and a yield exceeding 80%. The logarithmic reduction in the HCP content was 2.5, which is a value comparable to that typically obtained for ProtA [106].

As reported above, both precipitation and ATPS can be considered highly promising and attractive alternatives to chromatography for efficient mAb capture.

## 4. Polishing Step of mAB Purification

### 4.1. Critical Impurities

The mAbs solution obtained after the capture step is characterized by a high purity. However, due to the strict requirements of regulatory agencies, this purity is insufficient for the product to be used in medicine. Therefore, the next step in the mAb production process is polishing. In this step, less-abundant impurities are removed, including process-related impurities, i.e., HCP, DNA, free ProtA ligands, viral and product-related impurities including charge variants and aggregates [21,36,49].

The International Conference on Harmonization has defined HCPs as a critical impurity that affects the quality of the final product. There are no clearly established limits for HCP content in the final product, but it is generally accepted that their content should be less than 100 ppm [36]. Removing HCPs during the polishing stage is the greatest challenge because they constitute a group of proteins with diverse, often very different physicochemical properties. Furthermore, due to advances in USP that have resulted in increasingly higher mAb titers, the concentration of HCPs in the culture supernatant is also rising [107,108]. In addition to HCPs, other significant contaminants that must be reduced to levels of ppm or even ppb include DNA and leached ProtA ligands.

Furthermore, full-length IgG mAbs are prone to multiple post-translational modifications that result in product microheterogeneity. Microheterogeneity in the final product can lead to a decrease in the activity, immunogenicity, stability, and efficiency of mAb-based drugs [109,110,111,112]. The most common source of microheterogeneity is the formation of charge variants [112,113,114]. Some studies have shown a lack of interplay between the variant pI and pharmacokinetics [112,115,116]; however, several other studies have indicated a decrease in the therapeutic potency of acidic variants compared to the main and basic variants and an increase in the potency of basic variants compared to the main species [109,111,117,118]. Nevertheless, the influence of the charge variant composition on the functionality of a mAb-based drug is the individual characteristic feature of the mAb type. Therefore, the variant composition falls under the category of product quality attributes and must be maintained within a target defined for a particular product [119,120,121].

To meet the aforementioned purity requirements, the polishing stage typically consists of at least two chromatographic operations, which are mainly based on ion exchange chromatography (IEX). IEX is a suitable tool for the removal of most of the contaminants contained in post-capture mAb solutions, as well as for the adjustment of the composition of charge variants, where they can be separated according to differences in their isoelectric points [120,121,122,123].

In addition to IEX, hydrophobic interaction chromatography (HIC) or mix mode chromatography (MMC) is used, but to a lesser extent. The choice of technique depends on the properties of the mAb, the content and type of impurities in the solution provided from the capture step, and the required parameters of the final product [23,124,125]. Furthermore, in some cases precipitation may also be exploited to replace chromatography in the mAb polishing step. The downsides and upsides of the techniques used in the polishing step are summarized in Table 1.

### 4.2. Characteristics of Ion Exchange Chromatography (IEX)

The advantages of IEX include high adsorption capacity, high selectivity, and scalability. In AEX, positively charged groups in the form of weakly basic (e.g., diethylaminoethyl or dimethylaminoethyl) or strongly basic (e.g., quaternary aminoethyl) groups are used. CEX uses resins with positively charged groups, including strong cationites typically functionalized with sulfopropyl or sulfoethyl groups, and weak cationites based on e.g., carboxyl groups [23,126].

IEX can be performed in flow-through mode or bind-and-elute mode. In the first case, contaminants bind to the chromatographic resin, while the target protein flows through. In the bind-and-elute mode, the elution order is reversed. The isoelectric point of most mAbs is higher than 7; therefore, CEX purification is typically used in the bind-and-elute mode, whereas AEX is performed in the flow-through mode. The choice between flow-through mode and bind-and-elute mode depends on the properties of the mAb and the impurities [23,38,127].

The diversity of contaminants in mAb materials enforces the application of orthogonal IEX methods in which CEX and AEX are sequentially assembled to complete the polishing step. This allows the removal of both negatively charged and positively charged impurities. CEX-AEX sequences can also be used to isolate individual charge variants from prepurified mAb materials [128].

**Figure 4:**
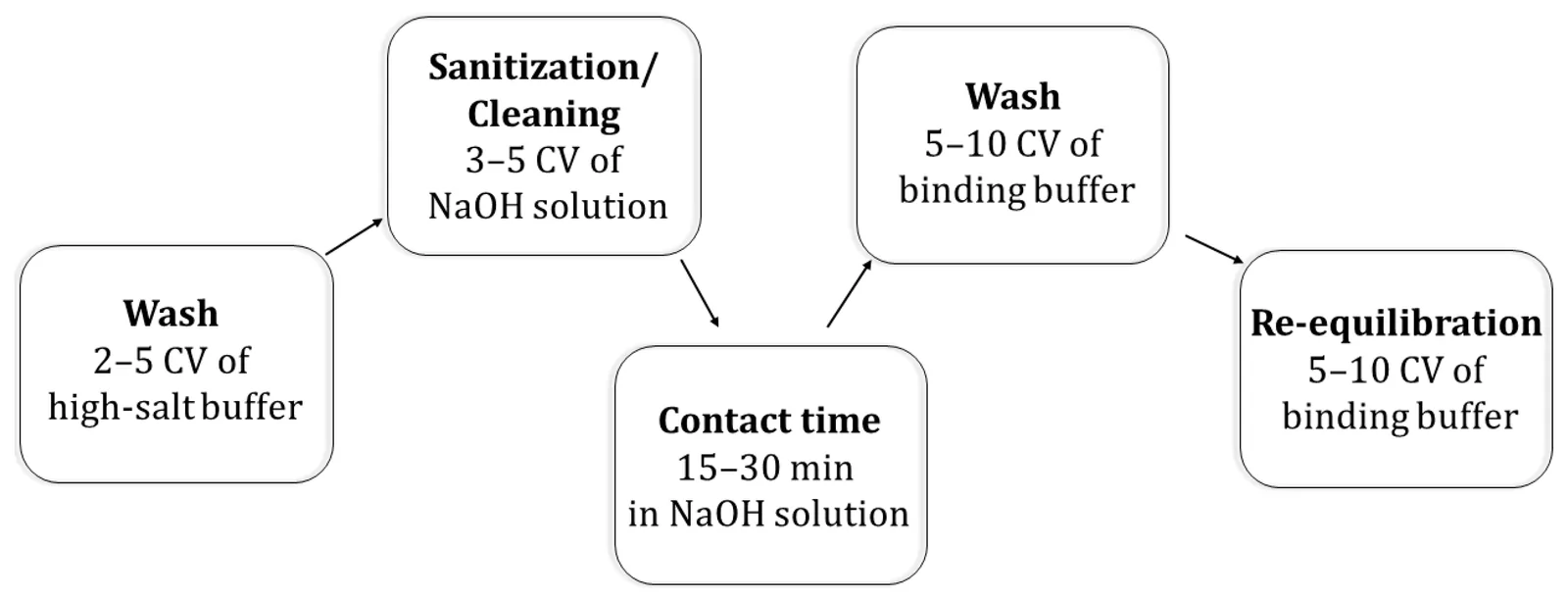
Typical CIP procedure for IEX resins.

In both flow-through and bind-and-elute modes, the process steps are the same: column equilibration, sample loading, wash, elution, column regeneration, and CIP. Loading is performed at low ionic strength and a pH that ensures an appropriate charge for the protein to binding to the resin. For the flow-through mode, throughput is usually higher than that in the bind-and-elute mode because the amount of mAb loaded is not limited by the bed adsorption capacity. Elution is induced by employing a salt or pH gradient. The salt gradient is achieved by increasing the concentration of NaCl in the mobile phase, while a pH gradient can be implemented externally by changing the buffer composition at the column input, or internally by chromofocusing using adsorbing buffer species or by the exchange of salt ions on weak acid or base groups on ion-exchanger matrices [127,129,130,131,132]. In some cases, a combination of both salt and pH gradient has been shown to be an efficient procedure for efficient mAb purification [131,132]. CIP is realized in a few subsequent steps in which the column is washed with high-salt buffers and NaOH solutions (Figure 4).

Despite its many advantages, IEX also has limitations: separation is possible only for charged particles; nonselective binding of contaminants cannot be avoided, and precise pH control is necessary. Additionally, the adsorption capacity of the resin depends on the ionic strength. Therefore, the use of additional buffer exchange techniques, such as membrane filtration prior to IEX, is very often required. Moreover, since both pH and salt composition alter the adsorption behavior of protein, the efficient realization of IEX requires robust process design based on the determination of the operating space of these parameters and sweet spots for which optimal process performance can be achieved. IEX can also involve specific effects that can adversely affect the separation efficiency, which are exemplified in Section 4.3.

### 4.3. Specific Effects Accompanying IEX

#### 4.3.1. Multiple-Peak Elution

**Figure 5:**
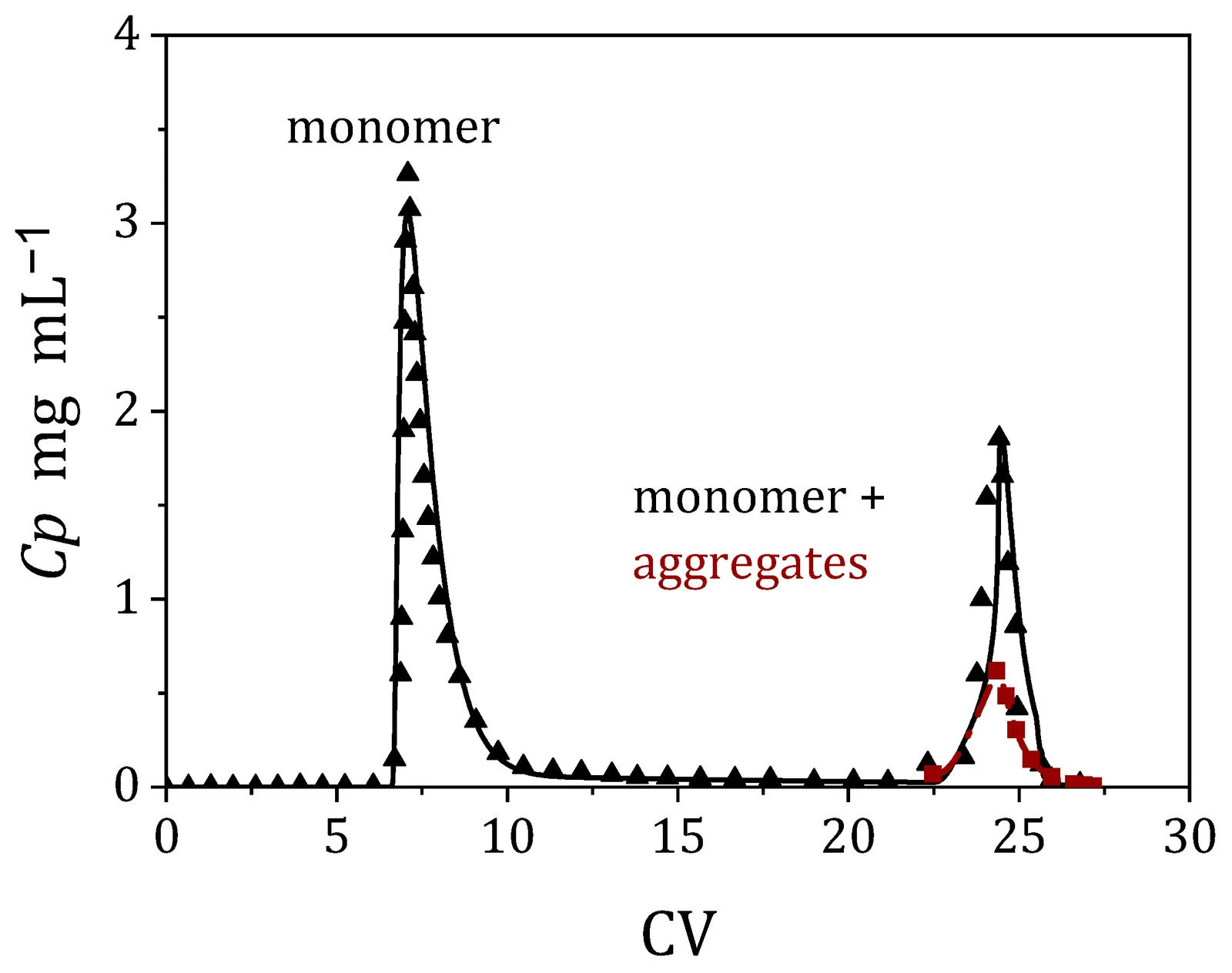
Illustration of the multiple peak elution of a mAb from an IEX column in a step pH gradient.

Separation in IEX is usually performed under mild conditions, which ensure stability of mAb structures. However, strong biding of the protein in the loading stage can trigger conformational changes in proteins upon adsorption. This phenomenon was also reported to occur for mAbs whose flexible structure is prone to destabilization [133,134,135,136,137]. It results in multiple-peak elution that is attributed to binding of the protein in different conformations [136,138] or associate formation that causes the protein to aggregate in column and consequently yield losses [134,139]. The phenomenon of two-peak elution is illustrated in Figure 5. The first eluting peak corresponds to the incomplete desorption mAb monomers while the second one, which is eluted at higher elution strength, corresponds to a mixture of aggregates and monomers that coexist in the adsorbed phase. The degree of aggregation is specific to the type of the protein and resin [136,140,141]. The selection of the resin and the binding conditions can be supported by a high-throughput method, in which a real-time polymerase chain reaction equipment is used to determine the temperature of the protein phase transition, i.e., the so-called melting temperature [142]. Changes in the melting temperature of the protein bound to the resin are screened under various adsorption conditions, and then correlated with the protein stability upon adsorption.

#### 4.3.2. Gradient Shape Deformation Due to pH Excursion

**Figure 6:**
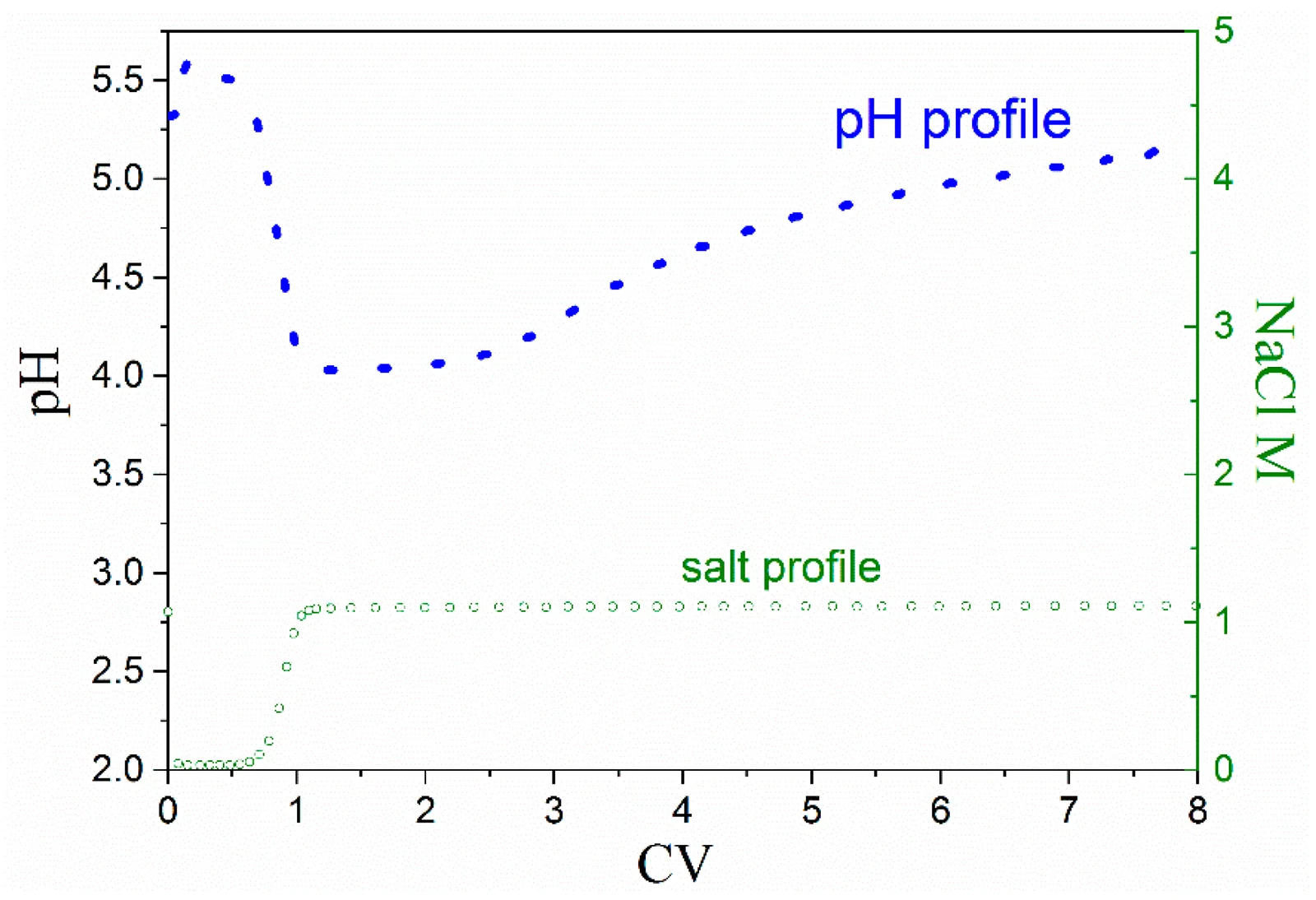
Illustration of the pH trajectory (top) triggered by a single-step change of the NaCl salt concentration (bottom) in acetate buffer on a weak CEX column (UNOsphere Tricorn).

The gradient shape is usually conserved during chromatographic elution on strong IEX resins. However, for weak ion exchangers a change in pH or salt concentration at the column inlet can trigger a pH excursion within the column that is caused by the exchange of salt ions on weak acid or base groups on adsorber functional groups [140,143,144]. An example of a pH excursion on a weak cation exchanger is illustrated in Figure 6. A potential issue with pH gradients can also be the pH excursion resulting from the dissociation of ionized groups of proteins adsorbed onto the chromatographic bed [137].

### 4.4. Alternative Chromatographic Methods for mAb Polishing

As mentioned above, in addition to IEX, hydrophobic interaction chromatography (HIC) or mix mode chromatography (MMC) is used in the polishing step.

HIC is a technique based on interactions between the hydrophobic regions of mAbs and the hydrophobic ligands of the chromatographic resin. The protein solution is loaded onto the column using solutions containing highly concentrated kosmotropic salts. Elution is performed at a reduced salt concentration. The need to use high salt concentrations during column loading and the complexity of the underlying hydrophobic interactions can induce protein conformational changes, which is the main limitation of this technique [143,145,146,147,148,149]. Several processes for purifying mAbs using HIC have been described in the literature. For example, Bresolin et al. used HIC with commercially available Toyopearl Phenyl 650 M (Tosoh Bioscience, Shunan, Japan) resin in the mAb polishing step. The process output was a mAb product with 99.9% purity at a yield of 96.2%. The levels of impurities such as aggregates, HCP, and DNA were below the detection limit for the analytical methods used [150].

MMC is classified as hydrophobic charge induction chromatography (HCIC), multimodal chromatography with a primary anionic ligand (MMAEX), and multimodal chromatography with a primary cationic ligand (MMCEX). This classification is related to the structure of the chromatographic column ligands. The literature contains many examples of successfully implemented MMC-based mAb purification processes [124,151,152,153,154,155]. The chromatographic resins used in this technique contain ligands that enable various interactions such as hydrophobic, electrostatic, hydrogen binding, and π-π interactions. By selecting appropriate process conditions, interactions can be limited to a single type or the synergistic effect of all of them can be utilized [124,140,156]. It allows for a broader range of separation selectivity for mAbs and contaminants [157]. Furthermore, MMC resins can also operate at higher ionic strengths, thereby reducing the need for buffer exchange [124]. The drawback of MMC is the high risk of protein unfolding and aggregate formation on the surface of the hydrophobic resin, as confirmed by a several studies [140,143,144,146,158]. Moreover, the combination of different mechanisms underlying adsorption results in an increase in the number of critical process variables to be determined in the process development stage.

### 4.5. Continuous Mode in the Polishing Step

Similarly to the capture step, attempts have been made to implement the continuous mode for the mAb polishing step. The general goal of these efforts is to develop integrated platforms that enable the continuous realization of the entire DSP. This solution has been described by Scheffel et al., who combined the ProtA capture step with a sequence of CEX and AEX chromatography [60,159]. The process was applied to the isolation and purification of pH-sensitive mAbs. It provided a pure antibody solution with HCP and DNA levels comparable to those of a typical industrial batch procedure. Furthermore, by using appropriate solvents and detergents during the inactivation step, the pH of the protein solution was maintained at 5.5, which significantly reduced the formation of protein aggregates [91,160]. The process was conducted in a periodic countercurrent chromatography system (PCC) consisting of three columns packed with CEX membranes. Other examples of continuous polishing steps operated in flow-through mode can also be found in the literature. Ichihara et al. proposed a combination of AEX and CEX that, by appropriately selecting the salt and pH gradient, could be operated in flow-through mode. The AEX-CEX setup was supplemented with activated carbon columns, which improved the reduction of HCP and DNA [161].

The continuous chromatography mode has also been used for the separation of charge variants. An example of such an application was provided by Anupa et al. [162]. That study presented an effective method for the separation of charge variants based on CEX, which allowed 99% recovery of mAbs, while simultaneously increasing resin lifespan and reducing buffer consumption.

### 4.6. Design of the Chromatographic Process

An indispensable element of the development of chromatographic purification is the design stage that is aimed at the determination of the operating space, in which the proper level of process quality attributes, e.g., throughput, yield, and product purity, is ensured. The operating space encompasses a combination of various process variables including column dimensions, column loading density, mobile phase flow rate, and the composition of buffers used for column loading, washing, and elution.

The design stage is handled by Quality-by-Design methods (QbD). The QbD strategies often exploit high-throughput microscale experimental methods that are used to derive data-driven models based on statistical analysis or machine learning algorithms [163,164,165,166,167,168]. The limited extrapolation capability of these methods frequently results in scaling up failure. A more efficient approach is based on knowledge-based design that exploits mechanistic modelling [169,170,171,172,173], or hybrid modelling, which is a combination of data-driven and knowledge-driven models [164,166,167,168]. A crucial element of these methods is mechanistic modeling.

Mechanistic models consist of differential mass balance equations that include kinetic and thermodynamic dependencies whose formulation requires the determination of a number of underlying parameters. Several mechanistic models have been developed to describe the dynamics of chromatographic columns [174,175]. The models differ in the formulation complexity, and the accuracy of their predictions depends on the proper determination of kinetic and thermodynamic coefficients [176,177]. Therefore, a tradeoff has to be found between the model overparameterization and its oversimplification. The combination of mechanistic modelling with statistical analysis allows the identification of critical process variables with the greatest influence on retention behavior, thus on the process quality attributes [178,179]. In general, in chromatographic separations, the dynamic loading capacity is the most critical variable, while in IEX the salt concentration and pH of the mobile phase also belong to the critical variables. Analyzing adsorption thermodynamics, i.e., shape of the adsorption isotherm, can also be an efficient tool that aids in the choice between different resins and elution mode [38]. In general, the design activity must include a quantitative description of the effect of pH and salt concentration on the relevant thermodynamic and kinetic parameters.

### 4.7. Nonchromatographic Technique for mAb Polishing

**Figure 7:**
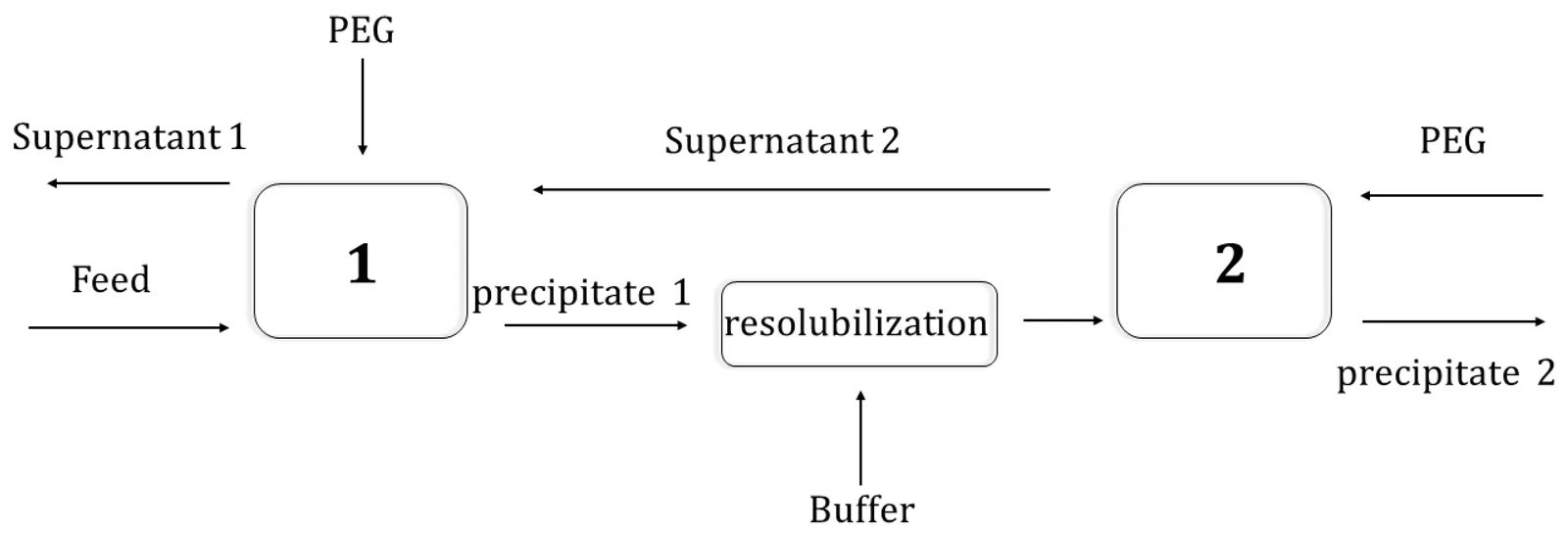
Scheme of countercurrent precipitation for adjusting the acidic variant content in a mAb pool. Feed and supernatants were transported countercurrently across the unit. Separation selectivity was adjusted by altering the PEG content in the solution [181].

Precipitation may also offer an alternative to eliminate or partially replace chromatography in the mAb polishing step. Rumanek et al. developed a multistage PEG-aided precipitation process for the reduction of the acidic variant content in a mAb pool. The authors showed that the presence of PEG in a low ionic strength solution favored precipitation of acidic variants. The process yield and the reduction of the acidic variant content depended on the number of process stages and on the composition of the pre-purified mAb solution. For example, processing a mAb material containing 42% acidic variants by two-stage countercurrent precipitation (Figure 7) allowed a 50% reduction in their content at a yield of 90%. To design the process, a mathematical model was formulated, which consisted of steady-state mass balance equations along with the underlying thermodynamic dependencies [180,181]. To improve the efficiency of this process, Zimoch et al. combined precipitation with AEX, which enhanced yield of the operation compared to the stand alone processes [128].

## 5. Formulation Step

### 5.1. Formulation Methods

The last stage of the DSP procedure is the formulation, which aims to ensure the appropriate composition of the final product. All mAbs currently approved are administered to patients intravenously. For this reason, mAb-based drugs are delivered to the market in liquid form or as a lyophilized powder that must be reconstituted in a suitable solution prior to administration. Selecting the appropriate form to ensure mAb stability is a factor of great importance. The formulation technique, as well as the form of the final product, should be properly selected to suppress processes that affect mAb activity. These include aggregation, fragmentation, and chemical modifications such as deamination and oxidation [113,182,183,184,185,186].

As most commercially available mAbs are sold in liquid form (Table 2), the predominant technique used for their formulation is a membrane-based process, such as ultrafiltration (UF) and diafiltration (DF) [187,188]. Their purpose is to exchange buffers to achieve the desired composition of the final product while preserving sterility of the solution. For a few mAb types that are sold in solid form, membrane filtration is coupled with freeze-drying. However, because the final product in solid form is characterized by greater stability and easier storage and transport compared to liquid formulation techniques, interest in solid formulations is growing. Therefore, an alternative formulation method has been proposed, such as crystallization [182,189,190,191,192].

The pros and cons of these formulation methods are provided in Table 1.

### 5.2. Liquid Formulation

As mentioned above, the predominant form of the final product for mAbs is a liquid. Therefore, the goal of a liquid formulation is to establish the appropriate composition of the solution. In the first formulation step, the purified mAb solution is concentrated by UF and then the buffer is exchanged using DF [187,194,195]. Both UF and DF utilize membranes that retain mAb molecules, while small molecules pass through the membrane pores. Because the ready-to-use mAb drug is administered by subcutaneous injection in a small volume, a high concentration of mAbs is required in the final product, often exceeding 100 mg mL^−1^ [183,184,191]. The requirement for highly concentrated mAb solutions makes membrane-aided formulation challenging. A high protein concentration is typically associated with high solution viscosity, which increases exponentially with increasing mAb concentration. To mitigate this issue, various stabilizers, e.g., arginine, were used. Unfortunately, even with their use, the high solution viscosity significantly reduces the efficiency of the membrane process. A high solution concentration induces concentration polarization, i.e., the deposition of molecules on the membrane surface, which results in a reduction in the permeate flow rate due to an increase in backpressure. High protein concentration also promotes fouling that is caused by blocking the membrane pores by deposited or adsorbed mAb molecules [182,187,188,189,196].

Typically, the pH of the final mixture ranges from 5.5 to 6.5, which is stabilized using an appropriate buffer; most commonly a histidine buffer. Sodium chloride is also sometimes used to achieve the desired osmolality and tonicity. Achieving the desired pH is not always easy during buffer exchange. Both the mAb molecule and the buffer components possess specific electrical charges. As a result, the Donnan effect occurs during filtration, which is defined as an uneven distribution of ions near protein molecules and across both sides of the membrane. This phenomenon leads to changes in the pH and concentrations of individual solution components, which significantly complicates the achievement of the desired parameters of the final product. Therefore, the Donnan effect has to be taken into account during the design of the filtration process. Numerous mathematical models capable of simulating and predicting this phenomenon have been described in the literature [92,182,197,198].

UF and DF are typically performed in batch mode in which, due to the relatively low conversion of commercially available membranes, the mAb solution is repeatedly recirculated. Prolonged processing time in combination with intensive mixing, pumping, and high protein concentrations often promotes mAb aggregation. To minimize this effect during both formulation and subsequent storage, surfactants are commonly added to the formulation. These compounds reduce protein adsorption at the gas–liquid interface and thereby limit aggregation phenomena.

The extended time for protein processing in batch mode is associated with significant economic costs and high buffer consumption. Consequently, current research efforts are increasingly focused on the development of continuous filtration techniques aimed at improving the efficiency of membrane-aided formulation processes. Such approaches are also highly relevant for the development of integrated platforms that enable fully continuous DSP. An example of a continuous filtration process has been described by Rucker-Pezzini et al. [199]. Their approach was based on a sequence of concentration and dilution steps performed using the Cadence Inline Concentrator (ILC) system (Cytivia, Marlborough, MA, USA), which allowed removal of more than 99% of impurities. However, the buffer consumption was approximately four times higher compared to conventional batch diafiltration. An improved solution is the Pall Cadence™ Inline Diafiltration system (Cytivia, Marlborough, MA, USA) designed for continuous single-pass tangential flow filtration (SPTFF). This technology requires less buffer than ILC, although its consumption remains higher than that in conventional batch DF processes. The advantage of the system is the simple process configuration, which requires fewer pumps than traditional batch setups. One of the major limitations of SPTFF processes is the progressive increase in mAb concentration, which increases the solution viscosity and thus generates backpressure exceeding the operational limits of the system. A potential solution to this problem has been proposed by Jabra and Zydney, who developed an optimized cascade configuration of membrane modules. In this approach, the feed stream was divided among multiple membrane cassettes, thereby reducing the retentate flow rate and limiting backpressure within the system [200]. Countercurrent continuous filtration systems have also been described in the literature. For example, Jabra et al. reported the concentration and buffer exchange of an IgG1 solution using a three-stage countercurrent diafiltration process integrated with the ILC system. The process allowed 99.9% impurity removal while maintaining the IgG1 recovery close to 100% [188,200].

The implementation of continuous membrane filtration processes into the formulation step requires advanced process control and automation strategies capable of maintaining stable product parameters over extended operating periods. Although process monitoring and control remain challenging, the literature describes several promising approaches based on PAT (Process Analytical Technology)-driven control architectures combined with continuous in-line monitoring of mAb concentration using near-infrared spectroscopy [201,202,203].

### 5.3. Solid Formulation

#### 5.3.1. Lyophilization

A considerably less common formulation method used in the biopharmaceutical industry is solid formulation. However, as mentioned above, due to the substantially greater stability of mAbs in the solid state compared to liquid solutions, this approach has gained increasing attention in recent years [6,190,204,205]. The predominant solid formulation is a lyophilisate obtained by freeze drying. According to Mieszkowski, approximately 7% of mAb-based products approved between 2021 and 2023 were freeze-dried products [184].

The freeze-drying process consists of three main stages: freezing, primary drying (removal of crystalline water under vacuum), and secondary drying (desorption of bound water). Among these, primary drying is the most time-consuming step. This is mainly associated with the requirement to maintain the process conditions below the collapse temperature (Tc), which is typically around 1–3 °C lower than the glass transition temperature of the concentrated solution subjected to lyophilization. A significant influence on the process is exerted by the presence of disaccharides such as sucrose and trehalose, which are commonly used as lyoprotectants. The glass transition temperature and, consequently, the Tc values for these compounds generally range from −28 to −32 °C. As a result, low pressure and shelf temperature conditions must be applied, leading to prolonged processing times [190,206,207,208]. Therefore, considerable efforts have been directed toward optimizing freeze drying in order to shorten the duration of primary drying [190]. One of the proposed strategies involves the application of microwave-assisted drying, which has been shown to significantly reduce primary drying time compared with conventional lyophilization methods [209]. Another approach is the use of combinations of crystalline and amorphous excipients, enabling the process to be performed more rapidly and at higher temperatures [208,210].

Despite continuous advances in lyophilization technology, freeze-drying remains a relatively complex, time-consuming and energy-consuming process. Consequently, more attention is being focused on the development of alternative formulation strategies that could be implemented during the formulation stage.

#### 5.3.2. Crystallization as an Alternative Formulation Strategy

Protein crystallization, which has been applied exclusively in crystallographic studies in the recent past, is increasingly being used in protein processing. The growing interest in this technique is due to its high productivity, operational simplicity, and relatively low operating costs. The crystalline protein phase is characterized by higher activity, purity, and stability compared to other formulations, which contributes to reduced bulk storage costs and a longer shelf life [34]. Furthermore, protein crystals can be used in injectable controlled-release systems. For the crystallization of mAbs, similarly to precipitation processes, PEG is commonly applied as a precipitating agent [6,7,204,211].

The major limitations restricting the broader implementation of crystallization in mAb manufacturing are the difficulty in determining suitable crystallization conditions and the challenges related to process scalability and its control. The literature reports studies in which crystallization conditions are selected based on the value of the second virial coefficient, B22 [212,213,214,215]. However, in practice, the development stage of protein crystallization is based primarily on the experimental determination of the operating window that is defined as the range of operating variables, i.e., the type and content of the precipitating agent, pH, and supersaturation level, for which the protein crystallizes [204,216,217,218,219].

In the next design step, the crystallization conditions are tuned, and the crystallization kinetics is determined in minicrystallizers, which are small vessels of approximately 1 mL. However, the hydrodynamic conditions in such small devices can differ significantly from those occurring in large-scale crystallizers. This excessively complicates the process scale-up, because hydrodynamic conditions strongly influence the characteristics and morphology of the resulting solid phase. To mitigate this issue and support the design of large-scale crystallization, Computational Fluid Dynamics methods can be used [220,221].

Several examples of successfully implemented mAb crystallization processes can be found in the literature. For example, Smejkal et al. presented a mAb crystallization process and its scaling from volumes of several milliliters to 1 L [20]. Huettmann et al. described the crystallization of a single-chain antibody in a two-phase system [222]. Zhang et al. purified mAbs using crystallization performed at the micro-batch scale [223]. In addition, templates have been employed to induce mAb crystallization. An example is the work of Rajoub et al., who presented template-assisted membrane crystallization [224].

**Figure 8:**
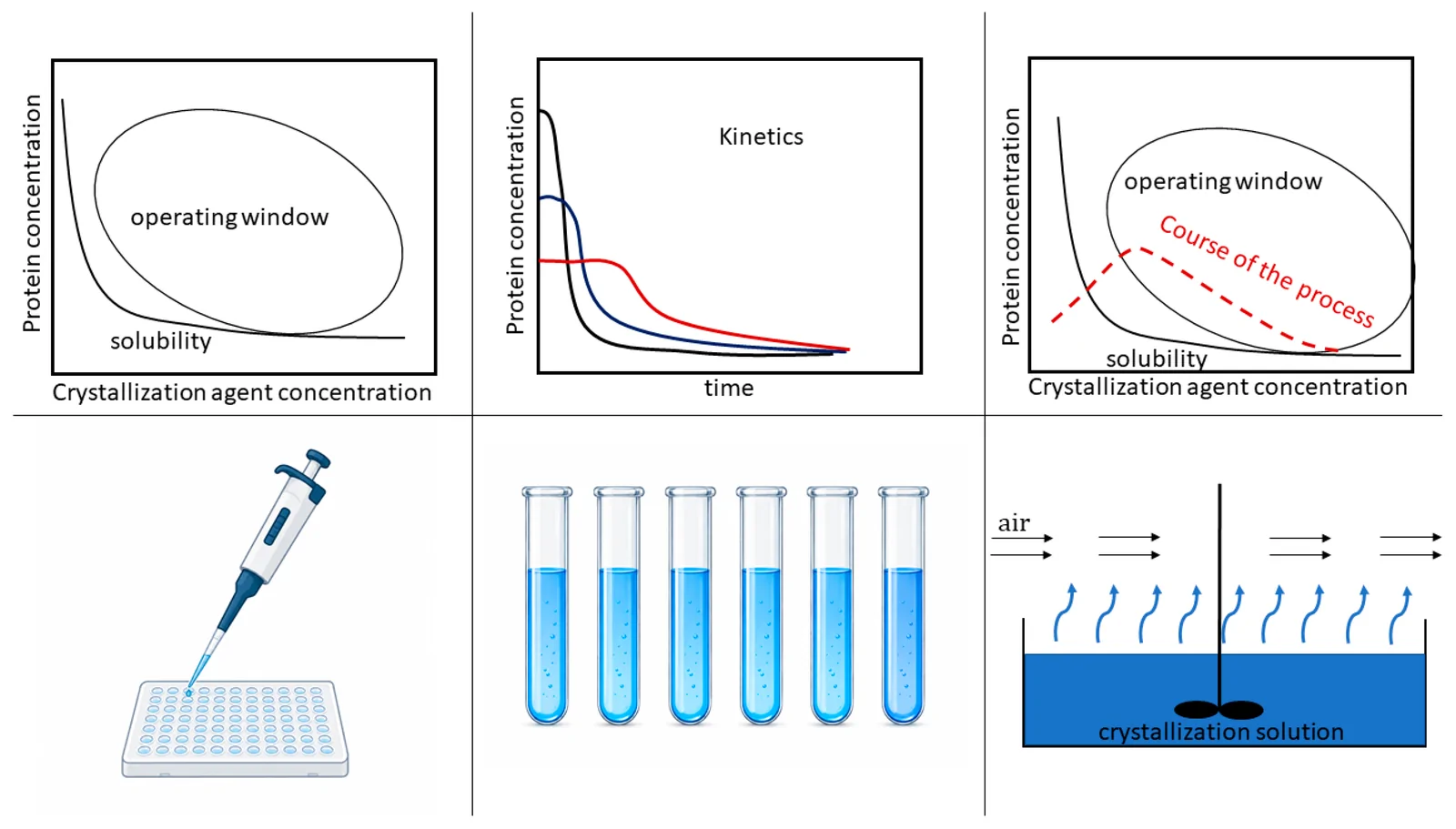
FCC design scheme: throughput screening to determine mAb solubility and the operating window for crystallization (**left**), measurement of the process kinetics in minicrystallizers (**middle**), predictions of FCC under the selected conditions, and process realization (**right**).

Kołodziej et al. developed a mAb crystallization process that was integrated with the sequence of two-stage precipitation and SLE illustrated above in Figure 3. The crystallization of mAbs was performed using forced convection crystallization (FCC), in which water was evaporated from the protein solution by forced convective air flow [6,225]. It allowed for controlled concentration of dilute protein solutions, which are typical for the capture and polishing stages, under mild conditions and at a high yield of 98–99% [6,225]. The obtained crystals exhibited high dissolution kinetics, which is a factor of major importance for the final form of drug administration [6]. The crystallization design procedure consisted of three stages: determination of the operating window by the throughput microplate method, measurement of the crystallization kinetics in minicrystallizers, and the realization of FCC with the aid of the determined process parameters (Figure 8).

## 6. Conclusions

Recent advances in upstream processing of mAbs have caused a substantial proportion of production costs to shift toward downstream processing (DSP). The standard DSP scheme consists of three main cost-generating and time-consuming operations: capture, polishing, and formulation of mAbs. Both the capture and polishing steps are predominantly based on chromatographic techniques, while formulation is accomplished using membrane-based technologies. Chromatography offers high separation selectivity that is indispensable to meet the restrictive demand for product purity; however, it suffers from relatively low productivity, complex equipment, and high operating costs. The latter is predominantly generated by protein A resins. Furthermore, the chromatographic polishing step involves material and buffer consuming procedures. Therefore, current research studies focus on the development of novel chromatographic resins, the improvement of elution mechanisms, and the implementation of continuous chromatographic processes to increase productivity and reduce material consumption.

Despite optimization of chromatographic operations, following the trend of ‘anything but chromatography’, new alternative nonchromatographic approaches for DSP applications are being developed including precipitation and aqueous two-phase extraction. In comparison to conventional chromatography, these techniques generally offer lower operating costs and higher process productivity. They may be especially effective in the capture step, where they can replace protein A chromatography. Furthermore, the application of precipitation and crystallization to polishing steps can also bring the benefit of a substantial reduction in material and buffer consumption.

Crystallization also appears to be a promising strategy for the formulation of mAbs. Protein crystals are significantly more stable and degradation-resistant forms than conventional liquid formulations. Furthermore, crystallization typically requires lower solvent consumption and can provide a higher productivity than membrane filtration, which is currently standard in the formulation stage. However, the determination of crystallization conditions remains a challenging task.

Furthermore, implementation of non-chromatographic methods is still restricted by a lack of rigorous platformization approaches. Nevertheless, intensive attempts that are made to solve that issue will reward in a wide industrial application of these methods in the near future.

In summary, the primary direction of development in mAb capture, polishing, and formulation technologies is the pursuit of more efficient and cost-effective solutions. Such innovations have the potential to reduce manufacturing costs, improve process sustainability, and ultimately expand patient access to advanced antibody-based therapies.

## Figures and Tables

**Table 1:** Assessment of downstream processing methods.

Methods	Advantages	Disadvantages	Development
Capture			
Protein A chromatography	High purity and yield, standardized workflow	High cost, ligand leaching, harsh elution conditions, limited caustic tolerance	Cost-effective and caustic tolerant ligands, new support materials, continuous mode
Precipitation	High yield, low costs, simple equipment	Low specificity, risk of aggregation and denaturation, lack of a platform	Efficient phase separation methods, platformization, continuous mode
Aqueous two-phase extraction ATPS	High biocompatibility, integrated processing, cost-effectiveness	Low specificity, difficult phase recovery, risk of aggregation	New solvents, centrifugal partition chromatography, continuous mode
Polishing			
Ion exchange chromatography	High resolution and selectivity, mild conditions	Sensitivity to buffer conditions, low productivity, high buffer consumption	Optimization of buffer conditions and solvent gradients, continuous mode
Hydrophobic interaction chromatography	High selectivity, workflow integration with IEX	Risk of aggregation, non-specific binding, low productivity, high buffer consumption	New ligands, optimization of solvent gradients, continuous mode
Mix-mode chromatography	High selectivity, high loading capacity	Low reproducibility, difficult optimization, high buffer consumption	New ligands, optimization of solvent gradients, continuous mode
Formulation			
Membrane filtration	High biocompatibility, high purity	Membrane fouling, high costs, limited lifespan of membranes, time-consuming process	New materials, fouling mitigation, flowsheet optimization, continuous mode
Liophilization	High stability of solid phase, rapid product reconstitution.	Time-consuming process, high equipment costs, high energy consumption	New lioprotectants, optimization of process parameters
Crystallization	High yield and purity, low costs, high stability of crystalline phase	Challenges in process optimization and design, lack of a platform	Continuous mode, new crystallization agents, platformization

**Table 2:** Antibody drugs approved by FDA (U.S. Food and Drug Administration) and EMA (European Medicines Agency) in 2024 and 2025 [1,193].

Molecule	Brand Name	Form	Concentration, mg mL^−1^
Donanemab	Kisunla™	liquid	17.5
Axatilimab	Niktimvo	liquid	50
Marstacimab	HYMPAVZI™	liquid	150
Crovalimab	PiaSky	liquid	170
Zolbetuximab	VYLOY	lyophilized powder	20 after reconstitution
Tarlatamab	IMDELLTRA™	lyophilized powder	~1 after reconstitution
Zanidatamab	Ziihera	lyophilized powder	50 after reconstitution
Zenocutuzumab	Bizengri	liquid	20
Odronextamab	Ordspono™	liquid	2
Garadacimab	ANDEMBRY^®^	liquid	167
Vilobelimab	Gohibic	liquid	10
Linvoseltamab	Lynozyfic	liquid	2
Nipocalimab	IMAAVY	liquid	185
Telisotuzumab vedotin	Emrelis	lyophilized powder	20 after reconstitution
Clesrovimab	Enflonsia	liquid	150
Sibeprenlimab	VOYXACT	liquid	200
Depemokimab	Exdensur	liquid	100
Narsoplimab	YARTEMLEA^®^	liquid	185

## Data Availability

The original contributions presented in this study are included in the article. Further inquiries can be directed to the corresponding authors.
